# Evaluation of surgical treatment strategies and outcome for cerebral arachnoid cysts in children and adults

**DOI:** 10.1007/s00701-024-05950-1

**Published:** 2024-01-27

**Authors:** Michael Schmutzer-Sondergeld, Aylin Gencer, Sebastian Niedermeyer, Stefanie Quach, Veit M. Stoecklein, Nico Teske, Christian Schichor, Nicole Angela Terpolilli, Mathias Kunz, Niklas Thon

**Affiliations:** Department of Neurosurgery, LMU University Hospital, LMU Munich, Marchioninistrasse 15, 81377 Munich, Germany

**Keywords:** Arachnoid cysts, Stereotaxy, Microsurgery, Hydrocephalus

## Abstract

**Objective:**

The best treatment strategies for cerebral arachnoid cysts (CAC) are still up for debate. In this study, we present CAC management, outcome data, and risk factors for recurrence after surgical treatment, focusing on microscopic/endoscopic approaches as compared to minimally invasive stereotactic procedures in children and adults.

**Methods:**

In our single-institution retrospective database, we identified all patients treated surgically for newly diagnosed CAC between 2000 and 2022. Microscopic/endoscopic surgery (ME) aimed for safe cyst wall fenestration. Stereotactic implantation of an internal shunt catheter (STX) to drain CAC into the ventricles and/or cisterns was used as an alternative procedure in patients aged ≥ 3 years. Treatment decisions in favor of ME vs. STX were made by interdisciplinary consensus. The primary study endpoint was time to CAC recurrence (TTR). Secondary endpoints were outcome metrics including clinical symptoms and MR-morphological analyses. Data analysis included subdivision of the total cohort into three distinct age groups (AG1, < 6 years; AG2, 6–18 years; AG3, ≥ 18 years).

**Results:**

Sixty-two patients (median age 26.5 years, range 0–82 years) were analyzed. AG1 included 15, AG2 10, and AG3 37 patients, respectively. The main presenting symptoms were headache and vertigo. In AG1 hygromas, an increase in head circumference and thinning of cranial calvaria were most frequent. Thirty-five patients underwent ME and 27 STX, respectively; frequency did not differ between AGs. There were two (22.2%) periprocedural venous complications in infants (4- and 10-month-old) during an attempt at prepontine fenestration of a complex CAC, one with fatal outcome in a 10-month-old boy. Other complications included postoperative bleeding (2, 22.2%), CSF leaks (4, 44.4%), and meningitis (1, 11.1%). Overall, clinical improvement and significant volume reduction (*p* = 0.008) were seen in all other patients; this did not differ between AGs. Median follow-up for all patients was 25.4 months (range, 3.1–87.1 months). Recurrent cysts were seen in 16.1%, independent of surgical procedure used (*p* = 0.7). In cases of recurrence, TTR was 7.9 ± 12.7 months. Preoperative ventricular expansion (*p* = 0.03), paresis (*p* = 0.008), and age under 6 years (*p* = 0.03) were significant risk factors for CAC recurrence in multivariate analysis.

**Conclusions:**

In patients suffering from CAC, both ME and STX can improve clinical symptoms at low procedural risk, with equal extent of CAC volume reduction. However, in infants and young children, CAC are more often associated with severe clinical symptoms, stereotactic procedures have limited use, and microsurgery in the posterior fossa may bear the risk of severe venous bleeding.

## Introduction

Cerebral arachnoid cysts (CAC) are benign cerebrospinal fluid (CSF)–containing lesions of the arachnoid mater and account for approximately 2% of all cerebral space-occupying lesions [4, 15, 16]. They are mainly diagnosed in childhood and young adulthood [39]. At the time of initial diagnosis, CAC can frequently lead to various symptoms depending on their localization, especially if a pronounced space-occupying effect is present [39]. However, due to the increasing number of cerebral imaging studies performed (cranial computed tomography (cCT) and cranial magnetic resonance imaging (cMRI)), the proportion of asymptomatic incidental cysts has increased particularly in adults [3, 38].

The indication for surgical therapy primarily considers the presence of neurologic deficits, noncommunicating hydrocephalus/signs of intracranial hypertension, and other symptoms due to the local space-occupying effect of the cysts, such as epileptic seizures. Furthermore, image morphologic characteristics such as ruptures, hemorrhages, or subdural hygromas are important in determining the indication for surgical therapy [6, 12, 26, 31]. Asymptomatic patients are usually followed up by cMRI imaging and not operated on. However, if symptoms—including atypical complaints such as vertigo, gait disturbance, or visual impairment develop during the course—the indication for surgery must be reevaluated.

Various approaches exist for the surgical treatment of CAC, such as microscopic/endoscopic cyst fenestration or resection, implantation of a cystoperitoneal or stereotactic internal shunt, or, in the case of concomitant CSF malresorption, implantation of a ventriculoperitoneal shunt [2, 7, 27, 30, 36]. The choice of surgical approach varies widely between centers because there are few prospective data on the optimal surgical approach, especially for different age groups [2, 27, 30]. Furthermore, to date, there are no studies evaluating postoperative symptom control according to each surgical approach.

In this study, we report our results in symptomatic, previously untreated CACs stratified by age groups (AGs) and the different surgical procedures. Special attention is given to periprocedural complications, outcomes, and risk factors for cyst recurrence.

## Materials and methods

### Patient population

After approval of the institutional review board of the Ludwig-Maximilians-University Munich (reference number 23–0348), the patient database of the Department of Neurosurgery was searched for all patients undergoing any surgical treatment of newly diagnosed cerebral arachnoid cysts between January 2000 and December 2022. Clinical and diagnostic evaluations were collected preoperatively and at routine follow-up evaluations (normally 3 months, 12 months, 24 months, and later). Functional outcome analyses referred to preoperatively obtained data. Patients were subdivided into three age groups: group 1 (age less than 6 years), group 2 (age 6–18 years), and group 3 (age older than 18 years). All patients and/or their parents gave informed consent before surgical treatment.

### Magnetic resonance imaging

According to our standard in-house protocol, the preoperative cMRI (1.5- or 3.0-T scanners: Magnetom Symphony, Siemens, Erlangen; Signa HDxt; GE Healthcare, Little Chalfont, UK) routinely included axial T2-weighted sequences (with slice thickness of 2 mm), 3-dimensional T1-weighted sequences before and after intravenous administration of gadopentetate dimeglumine (0.1 mmol/kg body weight; Magnevist; Schering Corporation, Kenilworth, NJ), and constructive interference in steady-state (CISS) sequences (with slice thickness of 1 mm), with axial, sagittal, and coronal reconstructions for each sequence. Volumetric cyst analyses of pre- and postoperative MR images were performed by semi-manual segmentation of pre- and postoperative T2 or CISS and contrast-enhanced (CE) T1 images using a commercially available software tool (SmartBrush®, Elements®, BRAINLAB AG, Munich, Germany). CAC in the middle cranial fossa were also subdivided into groups I-III according to the Galassi classification [10].

### Treatment protocol

**Fig. 1:**
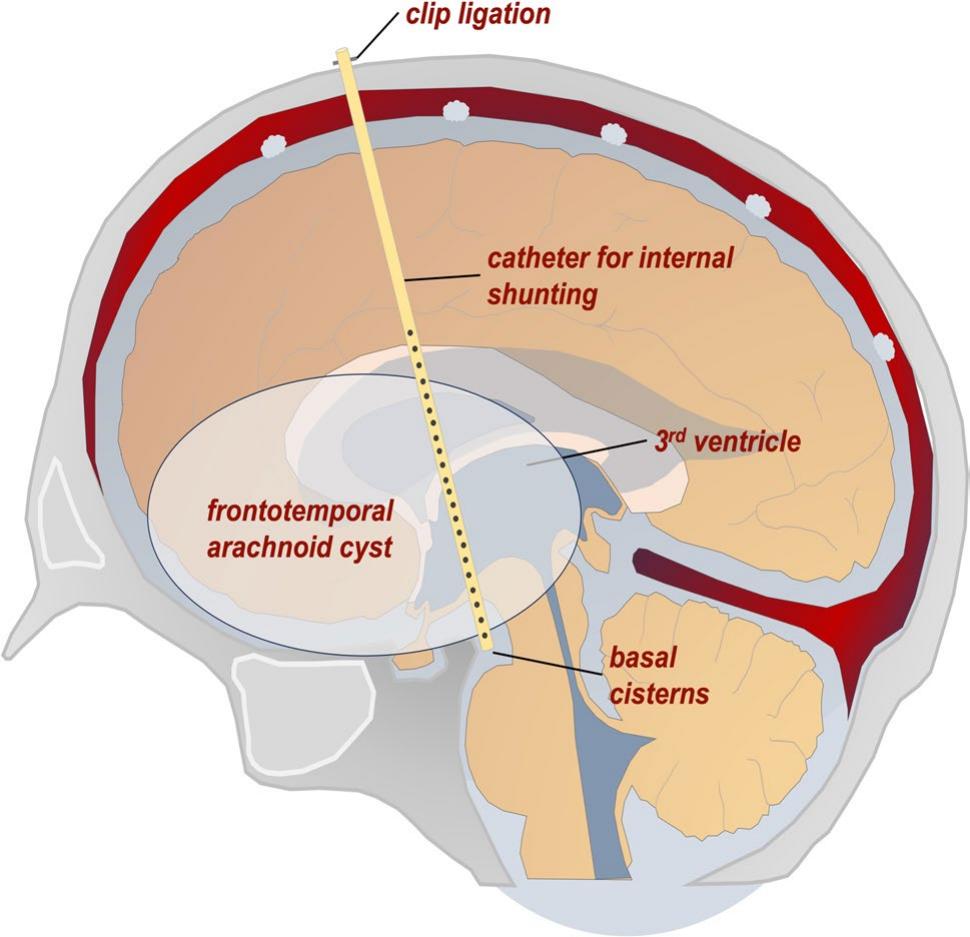
Schematic illustration of a stereotactically placed internal shunt catheter to achieve a permanent drainage of a large frontotemporal cerebral arachnoid cyst into both the ventricles and the basal cisterns

Indications for surgical therapy were refractory epileptic seizures, progressive headaches, or new-onset neurologic deficits such as paralysis or a percentile jump in head circumference in infants. Surgical interventions included microsurgical and/or endoscopic procedures (ME) to fenestrate or resect parts of the cyst wall to establish continuous drainage into the physiologic CSF pathways. For minimal-invasive treatment, an internal shunt catheter was implanted stereotactically (STX) to connect the cyst to the ventricular system and/or the basal cisterns depending on the individual CAC localization and configuration [40]. For the latter technique, surgical planning (iPlan stereotaxy; Brainlab, Munich, Germany) was based on a stereotactically localized contrast-enhanced computed tomography (CT) scan (0.6 mm slice thickness) and the preoperative MRI data (T1-weighted without contrast, T2-weighted/CISS sequences, contrast-enhanced magnetic resonance angiography), which were co-localized with the CT scan. A 1.3 mm diameter catheter (Becker EDMS ventricular catheter; Medtronic Inc, Dublin, Ireland) was stereotactically implanted via a 2-mm burr hole. Additional catheter perforations were added manually to achieve optimal up- and downstream drainage. The catheter was fixed extracranially with a hemoclip (Titanium Ligation-Clip, 150 mm length, B Braun, Melsungen, Germany) placed orthogonally on the catheter on the calvaria preventing the catheter from sliding into the brain. Above this, a sponge sealant patch (TachoSil®, Takeda Pharmaceuticals, Konstanz, Germany) was attached for adequate closure and additional fixation. A schematic illustration of the stereotactic implantation of an internal shunt for CAC drainage is shown in Fig. 1.

The preferred surgical approach was selected for each individual patient by consensus between experienced surgeons depending on the location of the CAC, cyst configuration, and individual risk assessment.

### Outcome analyses

Surgical results and follow-up analyses were assessed clinically and by quantitative MRI volume measurements. Clinical outcome data included perioperative complications and symptoms course. Recurrence was defined as any new cyst formation after complete excision, an increase by 25% in volume after partial relief, and/or new cyst-associated clinical complaints.

### Risk assessment

Perioperative morbidity rates were determined according to all documented medical, neurological, and approach-related adverse events. Transient and permanent deficits were differentiated.

### Statistical methods

The reference point of this study was the date of the first surgery. The last follow-up date was December 2022. The primary study endpoint was time to CAC recurrence (TTR). Secondary endpoints were outcome metrics including clinical symptoms, MR-morphological analyses, and complications. TTR was analyzed by using the Kaplan–Meier method. To compare the survival curves, the log-rank test was used. Results were tested by using a 2-way analysis of variance test (ANOVA), Student’s *t*-test, and Fisher’s exact test. For risk factor analyses, uni- and multivariate tests were conducted. GraphPad PRISM8.0d software was used for statistical analysis (GraphPad, San Diego, CA, USA). Statistical significance was as assumed at *p* < 0.05.

## Results

### Patient characteristics and preoperative symptoms

Sixty-two patients were included, of whom thirty-one were female (m:f = 1:1). Median age was 26.5 years (range, 0–81.6 years). Age groups (AG) less than 6 years (AG1), 6–18 years (AG2), and older than 18 years (AG3) included 15, 10, and 37 patients, respectively. The leading clinical symptoms to confirm the indication for surgery at the time of initial diagnosis were headache (24/62 patients, 38.7%), vertigo (11/62 patients, 17.7%), epileptic seizures (10/62 patients, 16.1%), nonspecific visual impairment (6/62 patients, 9.7%), and symptoms of increased intracranial pressure (ICP) (8/62 patients, 12.9%). In age group 1, the main presenting symptoms were an increased ICP (*p* = 0.006) and development of a macrocephalus with crossing of growth percentiles for the head circumference (*p* = 0.007), whereas in age group 3, headache (*p* = 0.07) and vertigo (*p* = 0.05) dominated. We documented a significant clustering of female patients among age group 3, while male patients tended to be more frequent in the younger groups (*p* = 0.003). There was a tendency of right hemispheric cyst localization in age group 3 (*p* = 0.2); temporal cysts were more common in groups 1 and 2 (*p* = 0.07). CAC of the posterior fossa tended to be more common in the older group (*p* = 0.4). Subdivision of middle cranial fossa CAC (*n* total = 25) according to the Galassi classification [10] did show a significant clustering of type III Galassi CAC (*p* = 0.01) in age groups 1 and 2 harboring large cysts with midline shifts and displacement of the temporal, frontal, and parietal lobe. The other CAC types according to the Galassi classification did not show any difference among age groups. In addition, preoperative hygromas (*p* = 0.005) and thinned cranial calvaria (*p* = 0.005) were significantly more frequent in group 1. Here, we also found a trend towards more ruptured cysts (*p* = 0.1). Initial CAC volume was smaller in group 3 (88.7 ± 86.0 cm^3^) compared to groups 1 and 2 (120.3 ± 80.1 cm^3^ and 145.7 ± 131.8 cm^3^, respectively) (*p* = 0.2). For further details, see Table 1.

**Table 1:** Patient characteristics and distribution of preoperative symptoms

Parameters	Age group 1 (0–6 years)	Age group 2 (6–18 years)	Age group 3 (≥ 18 years)	*p* value
Patient characteristics				
Total, *n* (%)	15 (24.2)	10 (16.1)	37 (59.7)	
Sex, *n* (%)				
Male	11 (17.7)	8 (12.9)	12 (19.4)	0.003
Female	4 (6.5)	2 (3.2)	25 (40.3)	
CAC side, *n* (%)				
Right	5 (8.1)	2 (3.2)	18 (29.0)	0.2
Left	6 (9.7)	7 (11.3)	14 (22.6)	0.2
Median	3 (4.8)	1 (1.6)	5 (8.1)	0.8
Bilateral	1 (1.6)	0	0	0.2
CAC location, *n* (%)				
Frontal	3 (4.8)	1 (1.6)	11 (17.7)	0.4
Parietal	0 (3.2)	2 (3,2)	4 (6.5)	0.2
Temporal	5 (8.1)	5 (8.1)	6 (9.7)	0.07
Occipital	0	0	2 (3.2)	0.5
Sellar	0	0	1 (1.6)	0.7
Posterior fossa	7 (11.3)	2 (3.2)	13 (21.0)	0.4
Cyst rupture, *n* (%)	2 (3.2)	2 (13.2)	1 (1.6)	0.1
Galassi type [10] (*n* = 25) (%)	n = 6	n = 7	n = 12	
I	1 (4.0)	0	4 (16.0)	0.2
II	1 (4.0)	1 (4.0)	4 (16.0)	0.6
III	4 (16.0)	6 (24.0)	4 (16.0)	0.07
Prominent optic sheaths, *n* (%)	11 (17.7)	2 (3.2)	13 (21.0)	0.01
Hygroma, *n* (%)				
Pre-op	8 (12.9)	3 (4.8)	4 (6.5)	0.005
Post-op	6 (9.7)	0	5 (8.1)	0.02
Expanded ventricular system, *n* (%)				
Pre-op	9 (14.5)	3 (4.8)	8 (12.9)	0.03
Post-op	3 (4.8)	0	3 (4.8)	0.2
Cranial calvaria thinning, *n* (%)	11 (17.7)	6 (9.7)	10 (16.1)	0.005
Initial volume (cm^3^)	120.3 ± 80.1	145.7 ± 131.8	88.7 ± 86.0	0.2
Follow-up, months	36.1 ± 27.5	41.3 ± 30.9	21.3 ± 19.1	0.02
Time to second surgery (recurrence), days	106.2 ± 66.5	319.0 ± 0	467.0 ± 716.1	0.4
Symptoms				
Symptoms pre-op, *n* (%)				
None	2 (3.2)	1 (1.6)	0	0.09
Paresis	1 (1.6)	1 (1.6)	2 (3.2)	0.9
Sensory disturbance	0	0	2 (3.2)	0.5
Headache	2 (3.2)	5 (8.1)	17 (27.4)	0.07
Epileptic seizures	2 (3.2)	3 (4.8)	5 (8.1)	0.4
Vertigo	0	1 (1.6)	10 (16.1)	0.05
Coordination disturbance	0	1 (1.6)	4 (6.5)	0.4
Increased ICP	4 (6.5)	1 (1.6)	0	0.006
Cranial nerve symptoms	1 (1.6)	0	2 (3.2)	0.7
Visual impairment	2 (3.2)	1 (1.6)	3 (4.8)	0.8
Mnestic disorder	1 (1.6)	0	2 (3.2)	0.7
Macrocephalus	3 (4.8)	0	0	0.007

### Surgical treatment

**Fig. 2:**
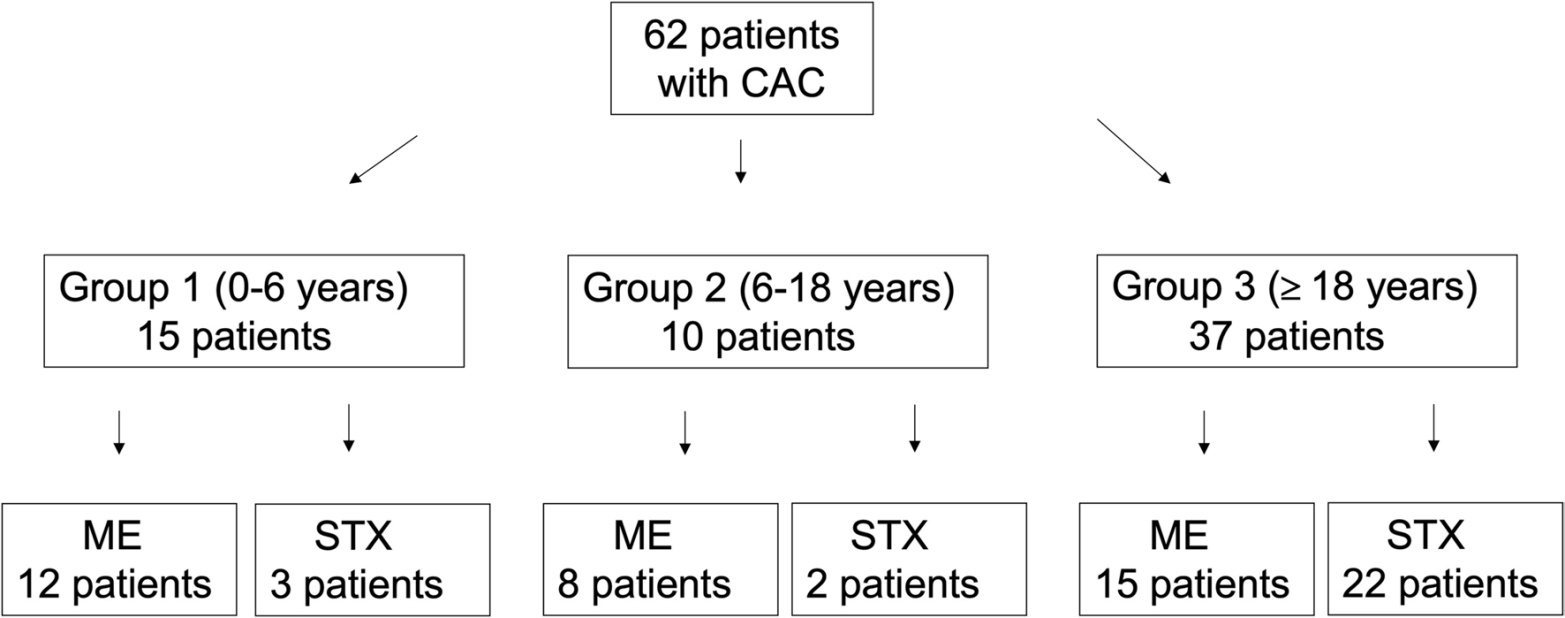
Consort diagram showing patient selection according to age groups 1–3 and treatment modality

From a total of 92 patients with cerebral arachnoid cysts included, 30 patients were clinically observed for asymptomatic cysts and 62 patients underwent surgery. Thirty-five patients underwent microsurgical/endoscopical cyst fenestration (age group 1, 12/15; age group 2, 8/10; age group 3, 15/37) and 27 patients stereotactical treatment (age group 1, 3/15; age group 2, 2/10; age group 3, 22/37) (Fig. 2). Further evaluation with respect to initial presenting symptoms and CAC locations showed no differences regarding treatment modality. For details, see Table 2.

**Table 2:** Distribution of patients’ symptoms and cyst location according to surgical therapy groups (*ME* microscopic/endoscopic fenestration, *STX* stereotactic implantation of an internal shunt catheter)

Parameters	Microsurgery/endoscopy, *n* = 35, %	Stereotaxy, *n* = 27, %	*p* value
Symptoms, *n* (%)			
None	1 (1.6)	2 (3.2)	0.6
Paresis	2 (3.2)	2 (3.2)	0.99
Sensory disturbance	0	2 (3.2)	0.2
Headache	15 (24.2)	9 (14.5)	0.6
Epileptic seizures	8 (12.9)	2 (3.2)	0.2
Vertigo	5 (8.1)	6 (9.7)	0.5
Coordination disturbance	3 (4.8)	2 (3.2)	0.99
Increased ICP	4 (6.5)	4 (6.5)	0.7
Cranial nerve symptoms	1 (1.6)	2 (3.2)	0.6
Visual impairment	4 (6.5)	2 (3.2)	0.7
Mnestic disorder	2 (3.2)	1 (1.6)	0.99
Macrocephalus	2 (3.2)	1 (1.6)	0.99
Cyst location, *n* (%)			
Frontal	9 (14.5)	6 (9.7)	0.99
Parietal	2 (3.2)	4 (6.5)	0.4
Temporal	10 (16.1)	6 (9.7)	0.8
Occipital	2 (3.2)	0	0.5
Sellar	1 (1.6)	0	0.99
Posterior fossa	11 (17.7)	11 (17.7)	0.6

### Outcome

**Fig. 3:**
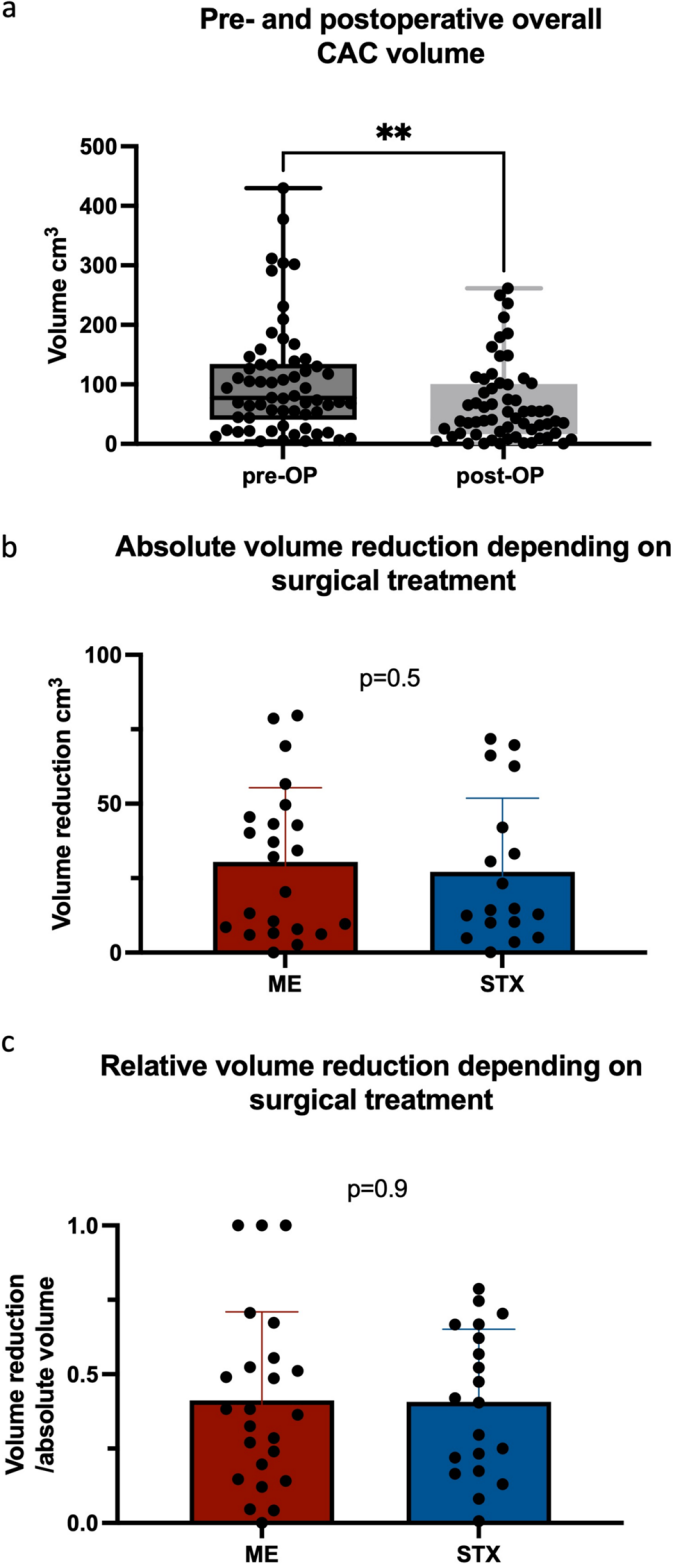
Pre- and postoperative cyst volume in the overall patient cohort (**p = 0.008, **a**) and absolute (*p* = 0.5, **b**) and relative (*p* = 0.9, **c**) volume reduction depending on surgical treatments microscopic/endoscopic CAC wall fenestration (ME) and stereotactic implantation of an internal shunt catheter (STX)

**Fig. 4:**
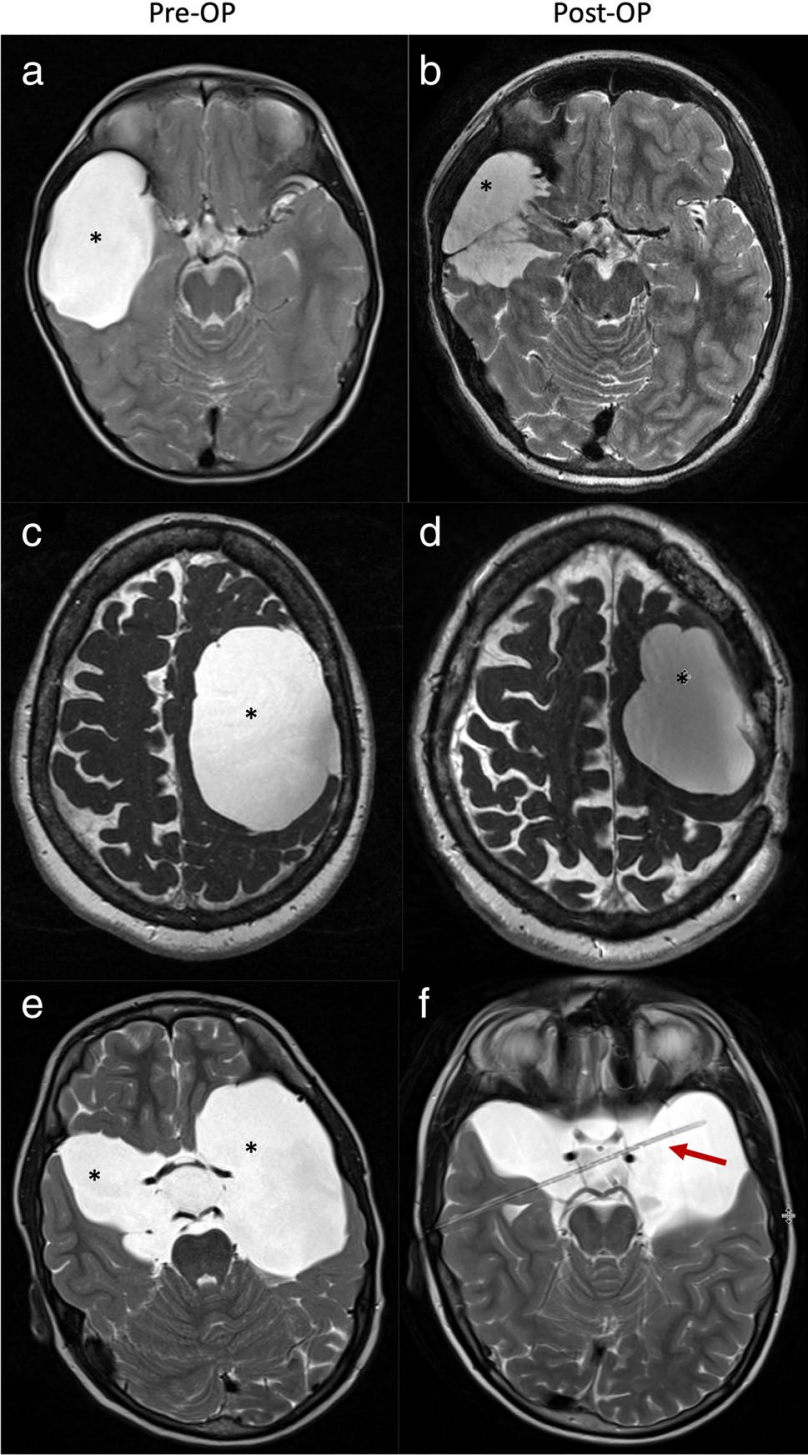
MR imaging of patients with cerebral arachnoid cysts (*) before (left) and after (right) microsurgical/endoscopic cyst wall fenestration (images **a** + **b** and **c** + **d**) and catheter implantation (images **e** + **f**) (red arrow). Volume reduction was achieved with all surgical approaches

Overall, therapy resulted in a significant reduction in median cyst volume after the first surgery from 77.2 to 40.3 cm^3^ (range, 0–249.3 cm^3^) (*p* = 0.008, Fig. 3a). The respective median volumes dropped from 77.9 cm^3^ (range, 5.9–311.6 cm^3^) to 54.1 cm^3^ (range, 0–261.1 cm^3^) after microsurgical/endoscopical fenestration (*p* = 0.1) and from 87.1 cm^3^ (range, 4.8–429.8 cm^3^) to 40.5 cm^3^ (range, 1.2–249.3 cm^3^) after stereotactical cyst drainage (*p* = 0.07). The extent of the absolute and relative final volume reduction did not differ between the two surgical groups (*p* = 0.5 and *p* = 0.9, respectively; Fig. 3b and c). MR imaging of CACs pre- and postoperatively after each surgical approach is shown in Fig. 4.

After surgical therapy, the overall cohort showed a significant improvement in preoperative headache (*p* < 0.0001) and CAC-associated dilatated ventricles (*p* = 0.004), regardless of the type of surgical approach. Furthermore, there was no significant difference in the improvement of the individual preoperative symptoms dependent on the surgical therapy (Table 3).

**Table 3:** Improvement of preoperative symptoms depending on surgical therapy

Symptom improvement	Microsurgery/endoscopy ratio of improvement, *n*, %	Stereotaxy ratio of improvement, *n*, %	*p* value
Paresis	1/2 (50.0)	1/2 (50.0)	0.99
Sensory disturbance	0	2/2 (100.0)	0.99
Headache	13/15 (86.7)	6/9 (66.7)	0.3
Epileptic seizures	2/8 (25.0)	0/2	0.99
Vertigo	2/5 (40.0)	5/6 (83.3)	0.2
Coordination disturbance	2/3 (66.7)	2/2 (100.0)	0.99
Increased ICP	3/4 (75.0)	4/4 (100.0)	0.99
Cranial nerve symptoms	0/1	2/2 (100.0)	0.3
Visual impairment	4/4 (100.0)	2/2 (100.0)	0.99
Mnestic disorder	2/2 (100.0)	1/1 (100.0)	0.99
Macrocephalus	0/2	0/1	0.99

### Treatment for recurrent cysts

**Fig. 5:**
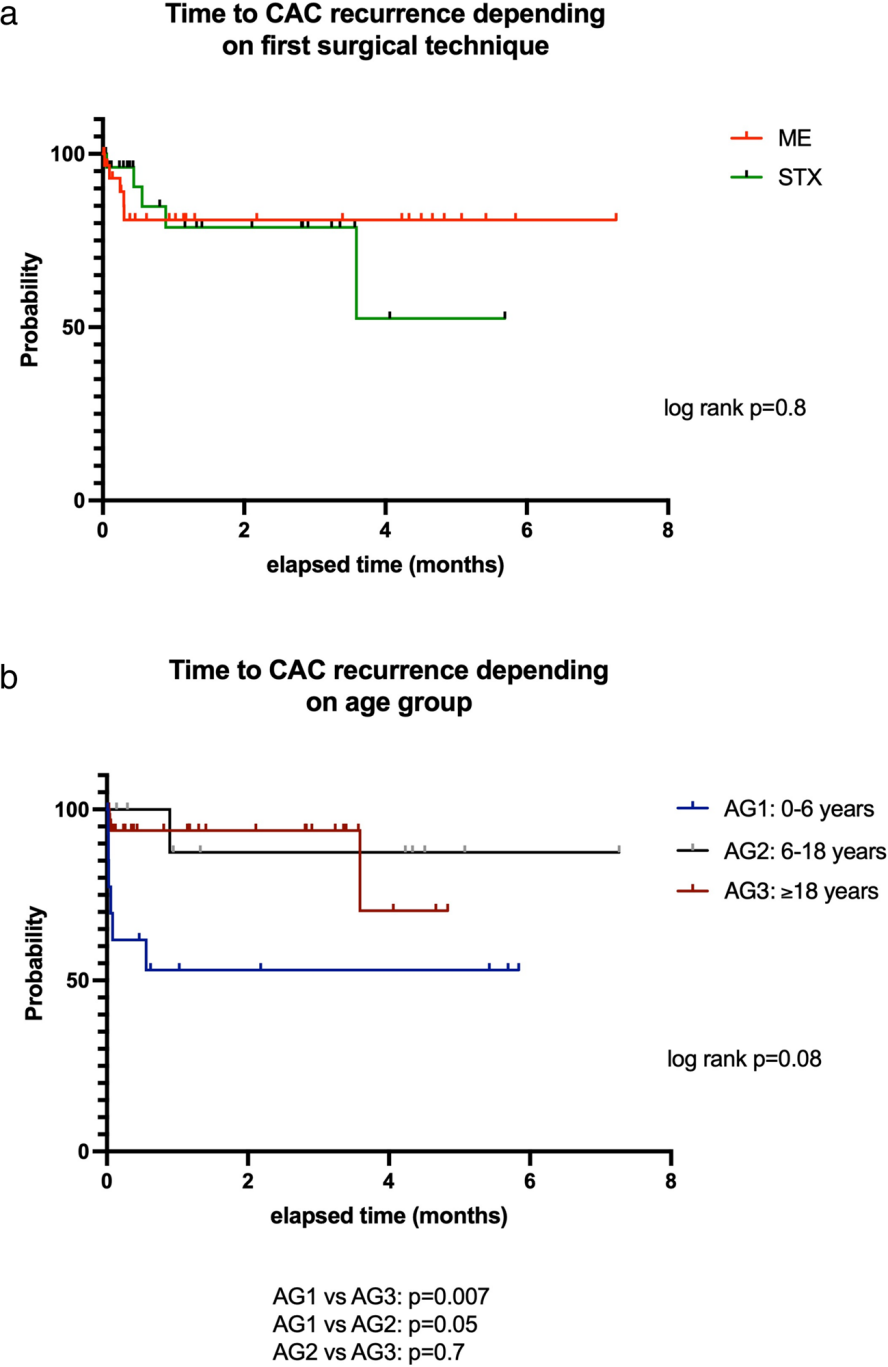
Time to second surgery for recurrent cerebral arachnoid cysts after microsurgical/endoscopical cyst wall fenestration (ME) and stereotactic implantation of an internal shunt catheter (STX) (**a**) and for the three age groups (**b**)

The median follow-up (FU) for all patients was 25.4 months but was significantly longer in age groups 1 and 2 (36.1 ± 27.5 months and 41.3 ± 30.9 months, respectively) as compared to group 3 (21.3 ± 19.1 months; *p* = 0.02). Overall, local CAC recurrence was noted in 10 patients (16.1%). CAC recurrences were associated with increased ICP (1/10 patients), new paresis (3/10 patients), coordination imbalance (2/10 patients), visual impairment (1/10 patients), and most often progressive headache (5/10 patients). Overall time to CAC recurrence (TTR) was 7.9 ± 12.7 months. The frequency of CAC recurrence did not differ according to the surgical procedure (5/35 (14.3%) microsurgery/endoscopy patients vs. 5/27 (18.5%) stereotaxy patients; *p* = 0.8). Median TTR was 3.5 months (range, 1.0–43.1 months) after microsurgery/endoscopy as compared to 5.2 months (2.9–10.6 months) after stereotaxy (log rank, *p* = 0.8, Fig. 5a). However, TTR between age groups 1 and 3 did differ significantly (*p* = 0.007). For details, see Fig. 5b. Recurrent cerebral arachnoid cysts were treated in 5 cases by cystoperitoneal shunt implantation and in 5 cases by re-fenestration. From the latter, 2 other re-recurrences were treated by fenestration again.

### Surgical morbidity

Overall, perioperative complications were seen in 9/62 patients (14.5%). These included severe complications in two infants (4 and 10 months old) with symptomatic brainstem compression due to multicystic CACs in the posterior fossa. In one case, a transient hemodynamically relevant bleeding from an atypical occipital sinus occurred during dura opening, and in the second case, a deep-seated, ultimately fatal venous hemorrhage occurred during an attempt at prepontine fenestration of a gross cyst wall with critical involvement of deep-seated neurovascular structures. Moreover, revision surgery was performed in 6 patients, due to a symptomatic hemorrhage into temporal cyst formation in 2 patients as well as significant CSF leakage despite careful dural closure in 4 patients after microsurgery/endoscopy. In the latter cases, revision surgery included an indication for ventricular-peritoneal shunting due to CSF malresorption. Complication after stereotactical catheter implantation was seen in one patient only developing postoperative bacterial meningitis despite antibiotic prophylaxis. The rate of complications after microsurgery/endoscopy with subsequent revision surgery tended to be increased (*p* = 0.06) compared to stereotaxy. Overall, a long-term shunt dependency as an indication of CSF malabsorption was seen in a total of 7 patients at 3.7 months (range, 0.7–10.6 months).

### Risk analysis for recurrent CAC

In univariate analysis, a preoperatively expanded ventricular system (*p* = 0.0006), paresis (*p* = 0.02), and patient age below 6 years (*p* = 0.005) were associated with recurrence of cerebral arachnoid cysts. Gender, cyst localization, and type of surgical approach did not influence the occurrence of cyst recurrence significantly. In multivariate analysis, age less than 6 years (age group 1) (*p* = 0.03), a dilated ventricular system (*p* = 0.03), and the presence of paresis (*p* = 0.008) were confirmed as significant risk factors for CAC recurrence (for details, see Table 4).

**Table 4:** Uni- and multivariate analysis for determination of risk factors for recurrent cerebral arachnoid cysts

Variable	Odds ratio	95% CI	*p*
A. Univariate analysis			
Age < 6 years	9.4	2.0–47.2	0.005
Sex (m vs f)	1	0.25–3.9	0.9
Hygroma pre-op	0.75	0.1–3.5	0.7
Hygroma post-op	4.3	0.9–19.4	0.06
Expanded ventricular system pre-op	13.3	2.9–96.7	0.0006
Expanded ventricular system post-op	3.0	0.4–18.3	0.3
Surgical approach			
Microsurgery/endoscopy	0.9	0.2–3.4	0.8
Stereotaxy	1.4	0.3–5.5	0.6
Ruptured cyst pre-op	1.3	0.06–10.4	0.8
Prominent optical nerve sheaths	2.4	0.6–10.4	0.2
Cyst localization			
Supra- vs. infratentorial	1.5	0.4–7.4	0.6
Side (right vs. left)	0.3	0.04–1.4	0.1
Pre-op symptoms			
Paresis	10.7	1.5–93.3	0.02
Focal deficits	2.8	0.9–28.7	0.06
Headache	0.6	0.1–2.6	0.5
Vertigo	0.5	0.02–2.9	0.5
Coordination disturbance	1.3	0.06–10.4	0.8
Visual deficits	1.0	0.05–7.6	0.9
Increased intracranial pressure	1.3	0.06–10.4	0.8
Macrocephalus	2.8	0.1–32.1	0.4
B. Multivariate analysis			
Age < 6 years	2.3	0.03–0.5	0.03
Expanded ventricular system pre-op	2.2	0.02–0.4	0.03
Pre-op symptoms			
Paresis	2.7	0.1–0.7	0.008

In addition, we performed univariate and multivariate analyses to determine risk factors for complications requiring revision surgery. Here, in the univariate data, preoperative (*p* = 0.001) and postoperative (*p* = 0.001) dilatation of the ventricular system and age less than 6 years (*p* = 0.007) were shown to be significant risk factors. In the multivariate analysis, only postoperative ventricular dilation (*p* = 0.02) was a risk factor for a complication requiring revision. Details are shown in Table 5.

**Table 5:** Uni- and multivariate analysis for determination of risk factors for surgical complications requiring revisions

Variable	Odds ratio	95% CI	*p*
A. Univariate analysis			
Age < 6 years	7.2	1.7–33.4	0.007
Sex (m vs f)	1.2	0.4–3.9	0.8
Hygroma pre-op	0.75	0.1–2.8	0.6
Hygroma post-op	2.1	0.5–8.3	0.3
Expanded ventricular system pre-op	7.4	2.91–28.8	0.001
Expanded ventricular system post-op	23.0	3.3–467.0	0.001
Surgical approach			
Microsurgery/endoscopy	1.4	0.4–4.9	0.5
Stereotaxy	0.8	0.2–2.7	0.7
Prominent optical nerve sheaths	2.4	0.4–4.2	0.2
Cyst localization			
Supra- vs. infratentorial	0.4	0.1–1.3	0.1
Side (right vs. left)	0.7	0.2–2.2	0.5
Pre-op symptoms			
Paresis	2.3	0.3–15.1	0.4
Focal deficits	1.3	0.2–6.8	0.8
Headache	1.1	0.3–3.5	0.9
Vertigo	0.3	0.01–1.6	0.2
Coordination disturbance	0.8	0.04–5.7	0.8
Visual deficits	0.6	0.03–4.2	0.6
Increased intracranial pressure	0.8	0.04–5.7	0.8
B. Multivariate analysis			
Age < 6 years	1.5	− 0.07–0.5	0.1
Expanded ventricular system pre-op	1.4	− 0.08–0.4	0.2
Expanded ventricular system post-op	2.4	0.08–0.8	0.02

## Discussion

This study reflects our findings in symptomatic cerebral arachnoid cysts with indications for surgical treatment in children and adults. We were able to include a comparatively large number of patients, collect clinical data on CAC in childhood and adults, present clinical and image morphologic outcome data, and report the risk of various surgical procedures. We could show that patients with symptomatic cerebral arachnoid cysts benefit from surgical treatment and exhibit long-term clinical improvement. Risk factors for cyst recurrence have also been identified. However, microsurgery/endoscopy was also shown to be associated with higher perioperative risk as compared to minimally invasive stereotactic procedures. This is especially true for infants with complex CAC localizations, where stereotactic procedures are not yet feasible due to the lack of ossification of the skull.

### Patient characteristics and symptoms

The patient characteristics of our study show that there was a gender balance in the overall cohort. Similar findings were reported by Spansdahl et al. [39]. We show, that Galassi type III cysts, preoperative hygromas, and a thinning of the cranial vault were significantly more frequent in infants and young children. The predominant symptoms leading to diagnosis and indication for surgery were refractory headache, epileptic seizures, dizziness, and nonspecific visual impairments which is in keeping with previous studies [27, 39]. Further, we divided our patient cohort into 3 age groups with a specific division into age group 1 (0–6 years), age group 2 (6–18 years), and age group 3 (older than 18 years). This was chosen due to the lack of an adequate possibility of the stereotactic catheter placement in very young patients (under 3 years) due to the lack of fixation options for the pins, so an age interval of up to 6 years was considered an adequate representation of the very young patient cohort, which has undergone both surgical treatment procedures equally (microscopy/endoscopy from 3 years of age). The further division into group 2 (6–18 years) and group 3 (older than 18 years) then emerged as a comprehensible classification to represent the other young patient cohort up to 18 years (*n* = 10) and adults (*n* = 37).

### Surgical treatment and outcome

Surgical therapy resulted in a significant reduction of headache symptoms in the overall cohort which is in line with previous reports [9, 41, 43]. In addition, other symptoms such as vertigo, coordination imbalance, and paresis also remitted postoperatively. Similar study results have been discussed previously [32, 33]. Notably, epileptic seizures did not ameliorate after surgery indicating preexisting alterations within the neuronal network that do not improve after cyst drainage. This has been reported earlier and must be considered with respect to treatment indications [28]. However, we also included 3 patients (age groups 1 and 2) without clinical symptoms. These patients had size-progressive CAC in follow-up MRI images, so surgery was indicated in order to prevent the onset of image morphological herniation with possible neurological symptoms. This is a particularly important point in the young patient cohort, as the children will experience an increase in size with possible neurological symptoms due to their young age. In the case of cysts with an image-morphologically pronounced brain displacement without an increase in size in follow-up MRI images, the entirety of clinical symptoms, eloquence, localization of the cyst, and possible ventricular displacement with consecutive hydrocephalus must be included in the indication.

Surgical treatment options showed a comparable reduction in cyst volume to a significant extent based on the overall cohort. Moreover, normalization of a preoperatively dilated ventricular system was seen. Notably, we did not observe a significant difference in volume reduction between the two surgical options. In contrast, other studies that compared endoscopic fenestration, resection, and shunting could describe a significantly better volume reduction after endoscopic cyst fenestration compared to resection and shunting [1, 14, 30]. In older patients, stereotactic catheter implantation was predominantly used in view of the increased perioperative risk associated with craniotomy. In addition, it must be mentioned that open cyst fenestration was performed in the case of morphological signs of cyst rupture or intracystic hemorrhage in order to have a sufficiently acceptable overview of the surgical field. Furthermore, the risk of catheter dysfunction after implantation in a hemorrhaged arachnoid cyst should be avoided. The stereotactical implantation of a catheter to drain intracerebral cysts has been specified and published in our and also other neurosurgical departments through years of experience and showed comparable effects in symptom improvement and complications compared to open cyst drainage [17, 22, 24, 34, 40]. We only used frame-based stereotactic catheter implantation in our cohort, so no statement can be made regarding frameless electromagnetic image-guided catheter implantation in our patient cohort. However, several studies [42, 44] have demonstrated the high precision and low risk of frameless neuronavigation for catheter implantation, which is comparable to stereotactic implantation, so this should also be evaluated in future studies. However, when assessing the success of surgical CAC treatment, the focus is rather on clinical than radiological criteria, since residual defect areas without clinical relevance due to congenital anomalies can persist after surgery.

### Cyst recurrences and revisions

CAC recurrences were necessary in about 16.1% of cases, independent of the applied procedure. Here, a TTR with 7.9 ± 12.7 months represents a rather short symptom-free interval. Cyst recurrences were treated by repetition of microsurgical fenestration or cystoperitoneal shunt placement. A far higher rate of surgery-requiring cyst recurrences was described in previous studies [5, 13]; therefore, sufficient cyst drainage can be assumed in most patients in our cohort. To define risk factors for recurrent CAC, a uni- and multivariate analysis was performed, which revealed a preoperatively extended ventricular system (*p* = 0.03), paresis (*p* = 0.08), and patients aged under 6 years (*p* = 0.03) to be associated with CAC recurrence. To our knowledge, this is the first study to define significant risk factors for recurrent cerebral arachnoid cysts, as other investigations [19, 20] only describe age and frontally localized extracerebral fluid collections being possible risk modalities for CAC recurrence. Ventricular dilation appears to be caused by the unilateral valve mechanism [11, 37] and an associated cyst enlargement due to CSF outflow obstruction. Highlighting preoperative ventricular dilation as a significant risk factor for recurrent cysts may alter future surgical strategies toward a single-stage combined procedure of fenestration or resection with additional ventriculoperitoneal or internal shunt placement, such that a second procedure need not be performed with delay. Also, paresis, which proved to be a significant risk factor in our study, has not yet been defined as such, even if it was only present in 6.4% of all cases preoperatively. However, focal neurological deficits, like paralysis, are an indication for surgery in size-progressive cerebral arachnoid cysts. This has already been confirmed in several studies [23, 35]. The presence of focal neurologic deficits should be given special consideration in surgical treatment planning, as the mass effect of CAC may cause greater adhesion of the cyst membrane to the arachnoid, increasing the risk of recurrence if fenestration or resection is insufficient.

The overall revision rate amongst our cohort was 14.5% with 12.9% after microscopical/endoscopical and 1.6% after stereotactic procedures and is in line with previous reports [17, 29]. Reasons for revision surgery were postoperative hematoma, CSF leakage, and postoperative bacterial meningitis during the course of the disease. All complications were managed and treated promptly by reoperation. However, in addition to revision surgery in adults, special attention should be paid to the young patient population. Two infants with large cysts in the posterior fossa and pronounced space-occupying effect with displacement of the brainstem and critical involvement of neurovascular structures had to be resuscitated because of noncontrollable intraoperative hemorrhage, in one case with fatal outcome. At this age, minimal-invasive stereotactic treatment of cerebral arachnoid cysts is often not feasible because of the lack of ability to fix the head in stereotactic frames, leaving only the option of microsurgical/endoscopic fenestration for large space-occupying cysts. Two other children who also underwent microsurgical/endoscopic fenestration had to undergo revision surgery after a mean of 18.5 days due to a new hydrocephalus or a CSF leak that developed secondary postoperatively, so the revision/complication rate in children after initial CAC seems to be remarkably high even in experienced hands. In univariate analysis to determine risk factors favoring revision, pre- and postoperative hydrocephalus (*p* = 0.001 each) as well as age < 6 years (*p* = 0.007) were confirmed as significant predictors. In the multivariate analysis, only postoperative hydrocephalus was significant (*p* = 0.02). Complications and frequencies after both microscopic/endoscopic and stereotactic procedures in the adult group of our cohort were comparable to other studies [2, 8, 18, 21]. In the pediatric cohort, we have to emphasize a high risk of intraoperative bleeding due to altered anatomical structures, which was also described earlier [25]. One study [18] defined a postoperative subdural hematoma as a possible risk factor favoring revision surgery; however, we were the first to describe postoperative hydrocephalus as a significant finding for re-surgery.

### Limitations

A major limitation of this retrospective study is the nonrandomized use of the available surgical strategies based on a case-based individual decision process by experienced neurosurgeons. Moreover, outcome data could only be drawn from pre- and postoperative time points at the last follow-up. In addition, the rather short follow-up period of this study must be emphasized and considered as a negative aspect. Due to the comparatively small patient number, possible bias cannot be excluded, especially in the multivariate analysis. In this respect, further prospective and even multicenter studies with a larger patient cohort may allow multivariate risk assessment. Beyond that, quality of life questionnaires, such as SF-36 (RAND 36-Item Short Form Survey) or EORTC-QLQ30, should be added to picture patient satisfaction and symptom relief/persistence in a realistic manner at least in adults, while the possibility of adequate quality of life mapping is lacking in very young patients.

## Conclusions

To our knowledge, this is one of the largest study cohorts comparing various surgical procedures for the treatment of cerebral arachnoid cysts. Microsurgic/endoscopic and stereotactic procedures proved to be safe and effective in volume reduction and improvement of symptoms at least in patients older than 6 years of age. The presence of a preoperatively expanded ventricular system, focal deficits like paresis, and a patient age under 6 years proved to be significant risk factors for recurrent cysts, which may be useful for future decisions on the most suitable surgical strategy by minimizing the need for several surgical procedures as well the risk of anesthesiology-related adverse effects.

## Data Availability

Informed consent was obtained from all individual participants included in the study.
